# Secondary metabolites, spectra characterization, and antioxidant correlation analysis of the polar and nonpolar extracts of *Bryophyllum pinnatum* (Lam) Oken

**DOI:** 10.5114/bta.2024.139752

**Published:** 2024-06-25

**Authors:** Chinyere E. Okafor, Ikechukwu K. Ijoma, Chiamaka A. Igboamalu, Chinaza E. Ezebalu, Chukwuemeka F. Eze, Jessica C. Osita-chikeze, Chisom E. Uzor, Adaugo L. Ekwuekwe

**Affiliations:** 1 Department of Pure and Industrial Chemistry, Chukwuemeka Odumegwu Ojukwu University, Uli, Nigeria; 2 Department of Pure and Industrial Chemistry, Nnamdi Azikiwe University, Awka, Nigeria

**Keywords:** spectrophotometry, correlation, ethnomedicine, phytochemical, absorbance

## Abstract

Oxidative stress-related pathologies have guided the scientific community into delving into natural product-based research on plant-based metabolites. Plant secondary metabolites serve as a valid alternative in managing oxidative stress-related pathologies. In this study, we present the secondary metabolite constituents of the polar extract (PE) and nonpolar extract (NPE) from the leaves of *Bryophyllum pinnatum*. These constituents were determined through qualitative and quantitative phytochemical screening. The functional groups and structures of these metabolites were determined based on FTIR and GC-MS experiments, respectively. Antioxidant and free radical scavenging (FRS) activities were determined using standard methods, including phosphomolybdenum, FRAP, DPPH, HRSA, and reducing power assays, with comparisons made to the ascorbic acid (AA) standard. Through Pearson correlation analysis, we estimated the relationship between antioxidant and FRS activities. The DPPH results revealed IC50s of 380.104 ± 0.001, 16.763 ± 0.001, and 7.684 ± 0.003 μg/ml for NPE, AA, and PE, respectively, indicating a trend of PE > AA > NPE. However, all other experiments showed a trend of AA > PE > NPE in antioxidant and FRS activities. These results showed the potential antioxidant and FRS properties of both PE and NPE. Additionally, the correlation analysis indicated a strong positive correlation between the antioxidant and FRS activities of PE and NPE. The research results suggest high antioxidant and FRS activities of PE and validate the use of *B. pinnatum* in managing free radical-related pathologies.

## Introduction

Plants have been extensively explored and researched for their medicinal relevance, including their antioxidant and radical-scavenging activities. Medicinal plants are highly esteemed for their beneficial therapeutic values due to the presence of secondary metabolites (Ijoma et al., 2023).

*Bryophyllum pinnatum* (syn. *Kalanchoe pinnata)*, also known as the miracle plant, is commonly referred to as Odaa opue (which translates to “it falls and grows,” indicating its resurrection properties) by the ethnic Igbos of Eastern and North Central Nigeria. This plant is classified under the family Crassulaceae. Previous research suggests the presence of secondary metabolites with pharmacological and pharmaceutical relevance, including essential oils (Mbachu et al., 2019). According to the literature review, compounds identified in *B. pinnatum* include bryotoxins, daigremontianin, bryophyllins, kalantubosides, bufadienolides, kaempferol, luteolin, etc. (Fürer et al., 2016; Abdulrahman, 2022; Yadav et al., 2022).

B. *pinnatum* is used in ethnomedicine for treating earaches, burns, abscesses, ulcers, insect bites, whitlows, diarrhea, immunosuppressive effects, memory-enhancing effects, and cithiasis (Fürer et al., 2016; Bhandari et al., 2021; Kassia et al., 2022). In traditional medicine, Bryophyllum species have been used to treat ailments such as infections, rheumatism, and inflammation (Nayak et al., 2010). It is also employed in African traditional medicine for remedies against otitis, headache, inflammations, convulsions, and general debility (Nguelefack et al., 2006; Oladejo, 2020), with reported antihistamine and antiallergic activities (Fürer et al., 2016; Yadav et al., 2021).

The use of *B. pinnatum* in treating premature labor and placental abruption by local midwives and ethnomedicinal practitioners in Southeast Nigeria has received scientific validation (Fürer et al., 2016; Santos et al., 2021). Due to their observed antimicrobial activity, lightly roasted leaves are externally applied to treat skin fungus (Imaobong et al., 2020; Moroof et al., 2022), while leaf infusions are used internally for fever treatment (Fürer et al., 2016). *B. pinnatum* is also used to expel worms, cure acute and chronic bronchitis, pneumonia, asthma, and other respiratory tract infections. Additionally, it is recognized as a sedative wound healer, diuretic, and cough suppressant (Fürer et al., 2016; Yadav et al., 2021). It finds application in treating kidney stones, gastric ulcers, and leg edema (Okwu and Nnamdi, 2011). Furthermore, *B. pinnatum* is widely used as an analgesic, carminative, and in managing nausea and vomiting (Majaz et al., 2011; Yadav et al., 2021).

Despite the numerous medicinal benefits of the assayed plant, few researchers have explored its ethnomedicinal benefits in managing and treating oxidative stress-related pathologies. Therefore, our research focused on its antioxidant and radical scavenging activities, along with analyzing the functional groups and compounds present in its secondary metabolites using FTIR and GCMS analysis, respectively. The research aimed to determine the secondary metabolites, antioxidant, and free radical scavenging (FRS) activities, as well as the functional groups and compounds in both the polar and nonpolar extracts of *B. pinnatum*. Statistical methods were employed to correlate the antioxidant and FRS activities of these extracts.

## Materials and methods

### Plant collection, identification, and authentication

The leaves were harvested in December 2022 from Bonsaac (6°10′58.5″N 6°43′39.2″E), Asaba, Delta State, Nigeria. The plant’s identification and authentication were performed by a botanist/taxonomist at the Department of Botany, Chukwuemeka Odumegwu Ojukwu University, Uli, confirming it as *B. pinnatum* (Lam) Oken (Voucher No. COOU/BTN/0062).

### Extraction

The fresh B. pinnatum leaves underwent drying in an electrothermal hot air oven (Eppendorf, Germany) at 35°C, followed by pulverization of the dried sample. The pulverized leaves were then weighed using an Ohaus balance (USA) and divided into two equal portions, each weighing 237 g. These two portions were stored in separate airtight containers for subsequent analysis. To the first portion, 2.0 l of methanol and H_2_O (1 : 1 v/v) were added and stirred for 2 h. Simultaneously, the second portion received 2.0 l of chloroform and underwent stirring for the same duration. Each mixture was then allowed to stand for 48 h, after which they were sieved separately using mucilin cloth and filtered through Whatman filter paper size No. 1. The filtrates from both portions were subjected to a hot water bath (Stericox, India) at 40°C to remove the extracting solvents, resulting in a crude concentrate of the extracts. The extract obtained with methanol–water was designated as apolar extract, while the chloroform extract was labeled a nonpolar extract. The percentage yield was estimated using Equation (1).


$$\text{Yield}[\%]=\frac{\text{Weight of extract}}{\text{Weight of pulverized plant sample}}\times 100$$ (1)

### Phytochemical screening

The extracts underwent qualitative and quantitative screening for tannins, saponins, flavonoids, alkaloids, steroids, terpenoids, glycosides, phenol, anthraquinone, resins, and phytochemicals, following the standard methods described by Ijoma et al. (2022). Additionally, phlobatannins were screened using the method described by Auwal et al. (2014). Each quantitative analysis was conducted in triplicate.

### In vitro antioxidant assays

#### Phosphomolybdenum reduction assay

The phosphomolybdenum method is based on the reduction of Mo (VI) to Mo (v) by the phytochemicals present in the extract, leading to the formation of a green phosphate/Mo (v) complex under acidic pH conditions (Ijoma et al., 2023). In this assay, 0.3 ml of the extract at varying concentrations (800, 400, 200, 100, and 50 μg/ml) and AA were mixed with 3 ml of a reagent solution (0.6 M sulfuric acid, 28 mM sodium phosphate, and 4 mM ammonium molybdate). This mixture was then incubated in an Eppendorf incubator (Germany) at 95°C for 90 min. After cooling to room temperature, the absorbance was measured at 695 nm using a spectrophotometer from Thermo Fisher Scientific (USA), with a blank used as the reference. The blanks consisted of methanol (0.3 ml) and 3 ml of reagent, while controls were prepared using 0.3 ml of H_2_O and 3.0 ml of reagent. The analysis was conducted in triplicate and results were expressed as the mean ± SEM.

The activity of the extracts is indicated by a percentage decrease in absorbance. The percentage phosphomolybdenum activity was estimated using Equation (2).


$$\text{Inhibition capacity}[\%]=\frac{\text{Sample absorbance }-\text{Control}}{\text{Sample absorbance}}\times 100$$ (2)

#### Reducing power assay

The extract’s reducing power was assessed following the method described by Oyaizu (1986), with slight modifications. This method relies on substances with reducing potential reacting with potassium ferricyanide (Fe^3+^) to produce potassium ferricyanide (Fe^2+^), which then reacts with ferric chloride to form a ferric–ferrous complex with a peak absorption at 700 nm. For each concentration (800, 400, 200, 100, and 50 μg/ml) of both the extract and standard, 1 ml was mixed with 2.5 ml of pH 6.6 phosphate buffer, and 2.5 ml of 1% potassium ferricyanide, followed by incubation at 50°C in a water bath for 20 min and subsequent cooling. Then, 2.5 ml of 10% trichloroacetic acid was added, and the mixture was centrifuged at 3000 rpm using an Eyla N-1000 centrifuge (Japan) for 10 min. Next, 2.5 ml of the supernatant was mixed with 2.5 ml of distilled water and 0.5 ml of freshly prepared 0.1% ferric chloride. The absorbance at 700 nm was measured against a blank containing only the reagents. This experiment was conducted in triplicate and results were expressed as the mean ± SEM. An increase in absorbance indicates the extract’s activity.

#### 2, 2-diphenyl-1-picryl-hydrzyl-hydrate (DPPH) radical scavenging assay

The extract’s DPPH FRS activity was evaluated following the method described by Brand-Williams et al. (1995). Approximately 0.5 ml of different concentrations (800, 400, 200, 100, and 50 μg/ml) of both the extract and standard were mixed with 3 ml of methanol and 0.3 ml of DPPH. The mixture was vortexed for 1 min and left to stand in the dark at room temperature for 30 min. Subsequently, using a spectrophotometer, the absorbance was measured at 517 nm against a sample blank containing 0.5 ml of the sample and 3.3 ml of methanol, with a control containing 3.5 ml of methanol and 0.3 ml of DPPH solution. The experiment was performed in triplicate and results were expressed as the mean ± SEM. The percentage DPPH was then estimated using Equation (3).


$$\text{DPPH scavenged}[\%]=\frac{\text{Control absorbance}-\text{Sample absorbance}}{\text{Control absorbance}}\times 100$$ (3)

#### Hydroxyl radical scavenging activity (HRSA) assay

The hydroxyl radical-scavenging activity of the extracts was measured using the method described by Jin et al. (1996), with slight modifications. In this method, hydroxyl radicals were generated through the Fenton reaction. These radicals are known to oxidize Fe^2+^ to Fe^3+^, and only Fe^2+^ can be combined with 1,10-phenanthroline to form a red complex known as 1,10-phenanthroline-Fe^2+^ with a maximum absorbance at 536 nm. Therefore, the concentration of hydroxyl radicals can be determined based on the extent of decolorization in the reaction solution. To summarize the procedure briefly, 1,10-phenanthroline solution (1.0 ml, 1.865 × 10^-3^ mol/l), phosphate-buffered saline (2.0 ml, 0.2 mol/l, pH 7.40), and extracts (1.0 ml of 800, 400, 200, 100, and 50 μg/ml) were sequentially added to a screw-capped tube and thoroughly mixed. Subsequently, a solution of FeSO_4_ · 7H_2_O (1.0 ml, 1.865 × 10^-3^ mol/l) was added to the reaction mixture. Following this, a solution of 1.0 ml of H_2_O_2_ (0.03% v/v) was introduced to start the reaction. The resulting reaction mixture was then incubated at 37°C for 60 min in a water bath, and the absorbance of the reaction mixture at 536 nm was measured against the reagent blank. For the negative control, the reaction mixture lacked any antioxidants, while for the blank, the reaction mixture was devoid of H_2_O_2_. The experiment was conducted in triplicate and results were expressed as the mean ± SEM. The percentage of hydroxyl radical (HR) scavenging activity (HRSA) was estimated using Equation (4).


$$\text{HRSA}[\%]=\frac{\text{Control absorbance}-\text{Sample absorbance}}{\text{Control absorbance}}\times 100$$ (4)

#### Ferric reducing antioxidant power (FRAP) assay

The method was based on Ijoma et al. (2023). In this method, 2 ml of fresh FRAP reagent, comprising 500 ml of acetate buffer (300 mM, pH 3.6), 50 ml of 2,4,6-tri(2-pyridyl)-s-triazine (TPTZ) (10 mM), and 50 ml of FeCl_3_ · 6H_2_O (50 mM), was mixed with various concentrations (800, 400, 200, 100, and 50 μg/ml) of each crude extract and AA. The corresponding optical density was then read after 180 s at 593 nm against the blank using a spectrophotometer. The analysis was conducted in triplicate and results were expressed as the mean ± SEM.

### Spectral characterization

#### Fourier transform infrared spectroscopy (FTIR) analysis

FTIR analysis was conducted to identify potential functional groups, using a resolution of 4/cm within the spectral range of 4000–400/cm. Ten (10) milligrams of dried *B. pinnatum* leaves PE and NPE were mixed with 100 mg of KBr salt pellet using a mortar and pestle, then compressed into a thin pellet to create a translucent sample disc. These powdered samples were then inserted into an FTIR spectroscope from Shimadzu (Japan).

#### Gas Chromatography Mass Spectroscopy (GCMS) analysis

The plant extract’s phytochemical constituents were characterized through GCMS analysis using a Varian 450-GC coupled with a 240-MS system from Varian (USA), equipped with an electron impact mode injector (70 eV) and a Varian data system. Mass spectrometry was utilized to identify the compounds in the GCMS chromatogram of *B. pinnatum* leaves PE and NPE. The interpretation of mass spectra peaks corresponding to unknown compounds involved matching them with a database of known compounds stored in the NIST library. Major components were identified using authentic standards obtained from computerized libraries.

### Statistical analysis

The statistical analysis was performed using SPSS version 21 for Windows. Results were expressed as the mean ± SEM. Differences among means were examined for statistical significance using a Tukey multiple comparison, complemented by a one-way analysis of variance (ANOVA). A *P*-value < 0.05 was considered statistically significant, and Pearson correlation was employed to ascertain the relationship between the antioxidant and radical scavenging activities of the PE and NPE of *B. pinnatum leaves*.

## Results and discussion

### Phytochemical screening

The weight of the methanol-water extract was 11.29 g, while that of the chloroform extract was 8.25 g, corresponding to a percentage yield of approximately 4.76% and 3.48%, respectively.

In this study, moderate quantities of saponins, tannins, flavonoids, and steroids were observed, while phenols were present in trace amounts in the PE. However, all other assayed phytochemicals in the PE were not detected at the concentrations analyzed. As for the NPE, glycosides, steroids, and flavonoids were present in trace quantities, while other phytochemicals were not detected (Table 1). The presence of phenols, flavonoids, terpenoids, and saponins, even in trace concentrations in the PE, suggests antioxidant and radical scavenging activities. Conversely, their absence in the NPE suggests that the PE is a superior radical scavenger and possesses better antioxidant activity than the NPE.

**Table 1:** Qualitative phytochemical screening of the PE and NPE of *Bryophyllum pinnatum* leaves

S/N	Secondary metabolite	Experimental method	PE	NPE
1	Saponins	Foam test	++	ND
2	Tannin (Catecholic)	Ferric chloride test	++	ND
3	Flavonoids	Magnesium–HCl test	ND	ND
		Zinc–HCl Test	ND	+
		Lead acetate test	++	+
		20% NaOH Test	+	ND
4	Alkaloids	Hagers test	ND	ND
		Wagner’s test	ND	ND
		Dragindroff test	ND	ND
		Marqus test	ND	ND
5	Steroids	Salkowiski test	++	+
		Libermans test	++	+
6	Terpeniods	Salkowski test	ND	ND
7	Glycosides	Keller–Kilani test	++	+
8	Phenol	5% FeCl_3_ test	+	ND
9	Anthraquinones	Borntragers test	ND	ND
10	Resin	Turbidity test	ND	ND
11	Phlobatannins	HCl test	ND	ND

(+) – present in trace concentration, (++) – present in moderately high concentration, (+++) – present in very high concentration, ND – not detected, PE – polar extract, NPE – nonpolar extract

**Fig. 1:**
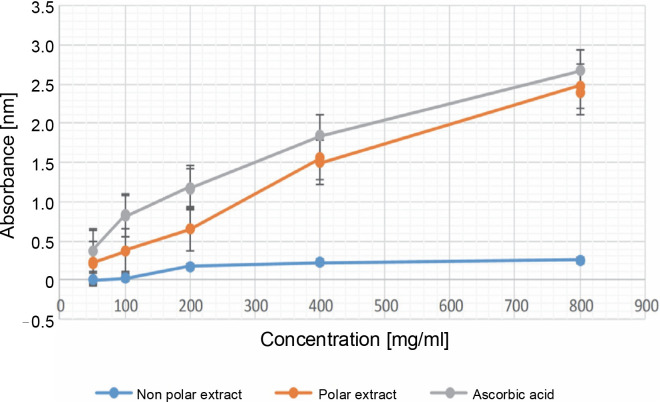
Absorbance in the reducing power antioxidant assay in 50, 100, 200, 400, and 800 μg/ml for both the NPE and PE of *Bryophyllum pinnatum* (Lam) Oken leaves extracts in comparison to AA; results were considered significant at (*P* < 0.05); results were significant at all the concentrations of the NPE, PE, and AA

**Fig. 2:**
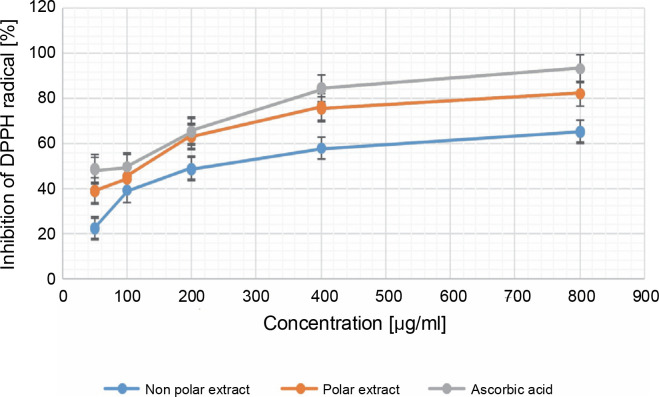
Percentage of inhibition in the DPPH radical scavenging assay in 50, 100, 200, 400, and 800 μg/ml for both the NPE and PE of *Bryophyllum pinnatum* (Lam) Oken leaves extracts in comparison to AA; results were considered significant at (*P* < 0.05); results showed no significant difference between concentrations of 50 and 100 μg/ml for AA

**Fig. 3:**
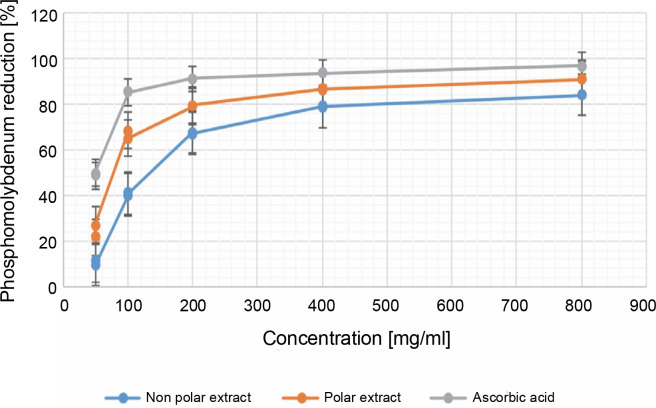
Percentage of inhibition in the phosphomolybdenum antioxidant assay in 50, 100, 200, 400, and 800 μg/ml for both the NPE and PE of *Bryophyllum pinnatum* (Lam) Oken leaves extracts in comparison to AA; results were considered significant at (*P* < 0.05); results showed a significant difference between all the extract concentrations of the NPE and all the concentrations of AA; however, there were no significant differences (*P* > 0.05) for PE at 200 and 400 μg/ml, and at 400 and 800 μg/ml

**Fig. 4:**
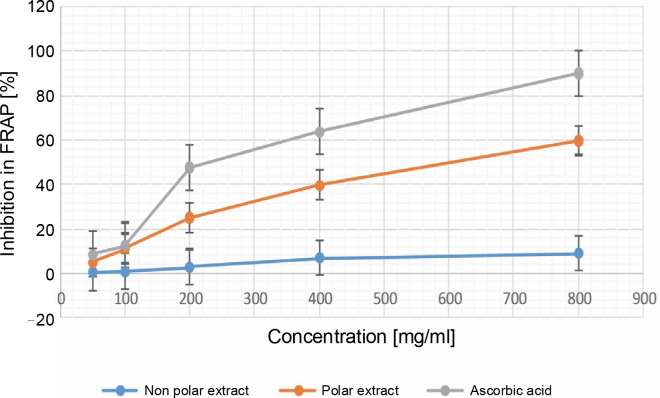
Percentage of inhibition in the FRAP antioxidant assay in 50, 100, 200, 400, and 800 μg/ml for both the NPE and PE of *Bryophyllum pinnatum* (Lam) Oken leaves extracts in comparison to AA; results were considered significant at (*P* < 0.05); results showed significant difference (*P* < 0.05) between all the extract concentrations of the NPE, PE, and the various concentrations of AA

**Fig. 5:**
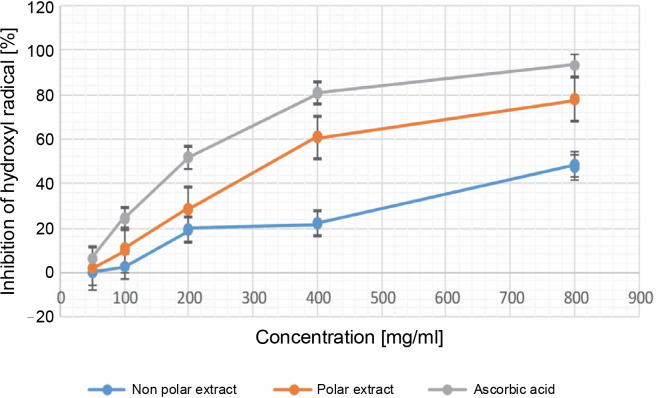
Percentage of inhibition in the HRSA assay in 50, 100, 200, 400, and 800 μg/ml for both the NPE and PE of *Bryophyllum pinnatum* (Lam) Oken leaves extracts in comparison to AA; results were considered significant at (*P* < 0.05); results showed a significant difference (*P* < 0.05) between all the extract concentrations of the NPE, PE, and the various concentrations of AA

The findings from the quantitative phytochemical screening of *B. pinnatum* leaves are shown in Table 2. The results suggest that the PE contained saponins, flavonoids, phenols, tannins, and glycosides at concentrations of 15.650 ± 0.250, 1.659 ± 0.052, 0.587 ± 0.042, 5.304 ± 0.077, and 1.329 ± 0.044%, respectively. This suggests enhanced antioxidant activity due to the presence of phytochemicals known for their strong antioxidant and FRS agents. The results of the quantitative phytochemical analysis validate the findings of the qualitative analysis, suggesting that due to the presence of phytochemicals with known antioxidant activity, the PE likely possesses better antioxidant and FRS activity compared to the NPE, as seen in Figures 1–5.

**Table 2:** Quantitative phytochemical screening of the PE and NPE of *Bryophyllum pinnatum* leaves

Sample	Alkaloids	Saponins	Flavonoids	Phenols	Tannins	Glycosides
PE	ND	15.650 ± 0.250	1.659 ± 0.052	0.587 ± 0.042	5.304 ± 0.077	1.329 ± 0.044
NPE	ND	ND	0.806 ± 0.092	ND	ND	ND

ND – not detected, PE – polar extract, NPE – nonpolar extract

Previous studies on isolating bioactive components from *B. pinnatum* using different solvents suggest that polar solvents like water, ethanol, and methanol are mostly suitable for extracting its bioactive constituents. The bioactivity of plant extracts largely hinges on both the extract concentration and the solvents used for extraction (Okele et al., 2019; Babatunde et al., 2023). These results corroborate our findings, indicating the higher bioactivity of PE and confirming that polar solvents are indeed mostly suitable for extracting bioactive constituents from *B. pinnatum*.

Our study indicated the occurrence of bioactive compounds such as flavonoids, phenols, alkaloids, saponins, and tannins, which align with the results of another study (Akacha et al., 2016; Bhandari et al., 2021). Phenolic compounds and those derived from phenols exhibit valuable antioxidant effects by scavenging reactive oxygen species (ROS) due to the presence of hydroxyl groups. Hence, quantifying phenolic content provides a clear understanding of a plant’s antioxidant potency and can thus predict its biological activities.

The greater phenolic content in the PE of *B. pinnatum* leaves (0.587 ± 0.042%) compared to its NPE (phenols not detected at coverage concentration), as reported in our study (Table 2), corresponds to the observed greater antioxidant activity in the PE. Additionally, plant-based flavonoids and phenolic compounds are recognized as free radical scavengers, thus contributing to the antioxidant activity of plants (Amiri, 2012; Bhandari et al., 2021).

Preliminary phytochemical screening is a very important step in the determination of various bioactive secondary metabolites in plants. These compounds play a key role in beneficial medicinal, therapeutic, pharmacological, pharmaceutical, and physiological activities, including antioxidant, antidiabetic, and anticancer activities (Bhandari et al., 2021).

The relationship between phytochemicals and antioxidant activity has been well established. Phytochemicals like phenols are recognized as potent radical scavengers with antioxidant properties (Jinxiang et al., 2020). Additionally, the antioxidant capacity of compounds such as saponins, flavonoids, terpenoids, tannins, steroids, etc. has been scientifically proven (Mooradian, 1993; Amarowicz, 2007; Chen et al., 2014; Gutierrez-del-Rio et al., 2021; Ijoma et al., 2023).

### Antioxidant analysis

#### Reducing power

Figure 1 showed the results of the reducing power assay. At a concentration of 50.000 ± 0.000 μg/ml, the absorbance values for NPE, PE, and AA were 0.00 ± 0.00, 0.217 ± 0.005, and 0.370 ± 0.008 nm, respectively. At the maximum concentration, the absorbance values were 0.135 ± 0.035, 2.435 ± 0.041, and 2.674 ± 0.003 nm for NPE, PE, and AA, respectively. These results indicate that PE exhibited superior reducing power compared to NPE. While AA demonstrated higher reducing power than PE, the reducing power of PE was comparable to that of AA. There was a significant difference (*P* < 0.05) observed between the various extract concentrations of NPE and PE, as well as between the various concentrations of AA. The reducing power of plant extracts has long been implicated as a measure of antioxidant capacity. The results obtained in this study revealed a trend of AA > PE > NPE in the reducing power assay. Additionally, Figure 1 showed that the reducing power of the extracts and AA was dose-dependent.

The reducing power assay also showed that, at various concentrations, PE exhibited superior reducing ability compared to NPE. However, AA showed better activity in the reducing power assay, probably due to its purity, in contrast to the crude extracts of *B. pinnatum* containing both antioxidants and pro-oxidants (Ijoma et al., 2023). These results align with the findings of Asiwe et al. (2021), who showed that *B. pinnatum* had a reducing capacity of 337.22 ± 26.98 μg/ml, representing a concentration producing an absorbance of 50%.

#### DPPH radical-scavenging activity

Figure 2 showed the results of the DPPH assay. At the minimum concentration, NPE, PE, and AA exhibited DPPH inhibitions of 22.306 ± 0.323%, 38.944 ± 0.108%, and 48.445 ± 0.392%, respectively. At the maximum concentration, these values were NPE (65.358 ± 0.272), PE (82.229 ± 0.117), and AA (93.315 ± 0.177), with corresponding IC50 values of 380.104 ± 0.001, 7.684 ± ± 0.003, and 16.763 ± 0.001 μg/ml, respectively. For NPE and PE, significant differences were observed between the various extract concentrations, whereas for AA, there was no significant difference (*P* > 0.05) between the concentrations of 50.000 ± 0.000 and 100.000 ± 0.000 μg/ml. The research showed a trend of PE > AA > NPE, indicating superior radical scavenging activity for PE. The significance of these results lies in PE from *B*. *pinnatum* being twice as effective as AA as a radical scavenger. Optimal antioxidant activity of *B. pinnatum* extract was produced at an 800 μg/ml concentration for both PE and NPE, compared to AA at the same concentration, which was observed to be significant (*P* < 0.05). The presence of phenolic compounds in PE, even in trace quantities, may account for its higher observed antioxidant activity compared to NPE.

Previous research showed that extracts and flavonoids from *B. pinnatum* showed antioxidant activity in the DPPH assay (Fürer et al., 2016); therefore, the higher flavonoid content in PE indicated higher antioxidant and FRS activities. Similarly, the leaf extract showed antioxidant activity in vivo on rats in a dose-dependent manner in the DPPH radical scavenging assay (Yadav et al., 2021), and a similar trend was also observed in this study in the DPPH assay.

#### Phosphomolybdenum

The phosphomolybdenum assay is used in the laboratory to estimate the total antioxidant capacity (TAC) (Ijoma et al., 2023). Figure 3 depicts the percentage inhibition of the phosphomolybdenum antioxidant assay. At 50 μg/ml, NPE, PE, and AA exhibited a phosphomolybdenum reduction of 10.410 ± 0.933, 24.469 ± 2.651, and 49.259 ± 0.450, respectively. At the maximum concentration, these values were 84.133 ± 0.030%, 91.040 ± 0.248%, and 96.814 ± 0.000% for NPE, PE, and AA, respectively.

There was a gradual increase in the percentage reduction of phosphomolybdenum by the extracts until reaching a plateau. However, at 800 μg/ml, AA showed the highest inhibition, closely followed by PE. Regarding NPE and AA, there was a significant difference (*P* < 0.05) at all concentration levels tested (i.e., 50–800 μg/ml). In contrast, for PE, there was no significant difference (*P* > 0.05) between concentrations at 200 and 400 μg/ml, as well as between 400 and 800 μg/ml. The results depict that as the extract quantity increases, there is a subsequent increase in reducing power. Thus, the trend observed in the phosphomolybdenum assay mirrored that observed in the reducing power assay, with AA > PE > NPE. PE showed greater TAC than NPE in the phosphomolybdenum assay. While AA showed slightly greater TAC than PE, their TACs were comparable at the maximum concentration (*P* < 0.05).

Compounds previously isolated from *B. pinnatum* have exhibited outstanding antioxidant activity, exceeding both AA and α-tocopherol (Fürer et al., 2016; Yadav et al., 2021). Hence, the greater antioxidant activity of AA compared to PE and NPE is likely due to its purity.

#### Ferric-reducing antioxidant power (FRAP)

The FRAP assay results are shown in Figure 4. The results suggest that at the minimum concentration, NPE, PE, and AA exhibited values of 0.215 ± 0.001, 4.818 ± 0.089, and 8.510 ± 0.125, respectively. Conversely, at the maximum concentration, the results were 9.024 ± 0.130, 59.761 ± 0.188, and 90.050 ± 0.063 for NPE, PE, and AA, respectively. Notably, PE displayed significantly higher FRAP activity than NPE, with PE being approximately 6.6224 times more potent in the FRAP assay. This trend of AA > PE > NPE in the FRAP assay further supports the robust antioxidant and FRS capabilities of *B. pinnatum*’s PE compared to its NPE counterpart.

At all of the tested extract concentrations (i.e., 50–800 μg/ml), the results revealed significant differences (*P* < 0.05) between the NPE and PE concentrations. A gradual increase in the percentage of inhibition was observed as the concentration of the extracts increased, indicating a dose-dependent pattern of inhibition.

The PE and NPE of *B. pinnatum* leaves produced increasing antioxidant activity as the concentrations of the extracts increased in the FRAP assay. However, AA showed better FRAP status compared to the extracts at various concentrations, a trend also observed in other studies (Al-Snafi, 2013; Yadav et al., 2021).

#### Hydroxyl radical-scavenging activity

Figure 5 depicts the results of the HRSA assays. The oxidative activity of the *B. pinnatum* leaf extract was evaluated using HRSA. It was observed that the NPE showed no activity at 50 μg/ml, and varying the dose further (even at the highest concentration, i.e., 800 μg/ml) did not result in an inhibition exceeding 50%. In contrast, the PE demonstrated an inhibition of 93.501 ± 0.000% at the maximum concentration, which was comparable to AA at the same concentration (Fig. 5). This showed the superior HR scavenging ability of the PE compared to the NPE.

Figure 5 shows the percentage inhibition of HR by the extracts and AA. The result showed that at 50 μg/ml, the inhibition of HR was 1.881 ± 0.171 and 6.157 ± 0.288 for PE and AA, respectively, while NPE did not exhibit HR inhibition at this concentration. At the maximum concentration of 800 μg/ml, the percentage inhibition of HR was 47.834 ± 0.628, 77.879 ± 0.228, and 93.501 ± 0.000% for NPE, PE, and AA, respectively. The results across different concentrations for both the extracts and AA were significantly different (*P* < 0.05) from each other. The extracts demonstrated dose-dependent inhibition of HR, indicating that higher extract concentrations correlated with greater inhibitory activity. The computed IC50 for PE and AA was 448.527 ± 0.001 and 298.143 ± 0.00, respectively. This showed that AA exhibited superior HR inhibitory activity compared to PE. Notably, NPE did not show inhibition above 50% at the maximum concentration, thus was not computed. Additionally, the HRSA assay exhibited a similar trend in radical scavenging and antioxidant activities as observed in the HR inhibition assay.

The reduced HRSA of the NPE differed from the findings reported by Asiwe et al. (2021). Their study showed that the ethyl acetate extract of *B. pinnatum* exhibited a higher HRSA of 107.45 ± 5.37 μg/ml. This variation could likely be attributed to the differing polarities of the extraction solvents.

### Correlation analysis

The results of Pearson’s correlation analysis regarding the PE and NPE of *B. pinnatum* leaves are shown in Table 3. The results showed a strong positive correlation between antioxidant activity and FRS activity for the NPE (*P* < 0.01). Similarly, for the PE, similar observations were observed; however, the correlation between reducing power and phosphomolybdenum was notably strong and significant at a *P*-value < 0.05. Our results suggest that in both the NPE and PE, antioxidant activities and radical scavenging activities exhibit a strong positive correlation. Therefore, irrespective of the solvents used in extracting the antioxidant and radical scavenging constituents from *B. pinnatum* leaves, the antioxidant and FRS activities of both PE and NPE consistently maintain a strong direct proportional relationship.

**Table 3:** Pearson’s correlation coefficients for the analysis of the NPE (not bolded) and the PE (bolded) of the leaves of *Bryophyllum pinnatum*

Radical scavenging and antioxidant activities	DPPH	FRAP	HRSA	Phosmolybdenum assay
Reducing power	0.948 ** 0.925 **	0.930 ** 0.985 **	0.907 ** 0.978 **	0.956 ** 0.732 *
DPPH		0.925 ** 0.969 **	0.902 ** 0.980 **	0.986 ** 0.871 **
FRAP			0.937 ** 0.990 **	0.876 ** 0.812 *
HRSA				0.852 ** 0.815 **

** correlation is significant at the 0.01 level (2-tailed),

* correlation is significant at the 0.05 level (2-tailed)

The strength of the association in a correlation analysis was described by Campbell (2021) as follows: very weak (0.0–0.19), weak (0.20–0.39), moderate (0.40–0.59), strong (0.60–0.79), and very strong (0.80–1.0). Hence, the relationship between antioxidant and FRS parameters were considered strongly positive, indicating a direct proportional relationship between these parameters for both PE and NPE. A similar trend was also observed for similar parameters elsewhere (Najafabad et al., 2014). Therefore, even though the PE showed greater antioxidant and FRS activity compared to the NPE, their relationship in terms of the trend in antioxidant and FRS activity remains consistent.

ROS scavengers generally describe any molecule of chemical or biological origin capable of detoxifying one or more ROS targets through various mechanisms defined by the structure and chemistry of both the ROS scavenger and the ROS targets (Herb and Schramm, 2021).

Therefore, a ROS scavenger targets both radicals (e.g., HR and oxygen radical) and nonradicals (e.g., H_2_O_2_), or multiple ROS targets, depending on the chemical structure of the scavenger. However, antioxidants encompass molecules or atoms capable of reducing an oxidizing substance and are not limited to ROS (Gutteridge and Halliwell, 2010; Poljsak et al., 2013; Herb and Schramm, 2021). Considering this definition, antioxidants may include not only ROS scavengers but also other chemicals that reduce reactive nitrogen species (Herb and Schramm, 2021). Therefore, an antioxidant does not literally translate to a ROS scavenger, and these terms should not be confused. Much like ROS probes, numerous commercially available ROS scavengers exist; however, they differ in structure and specificity toward ROS targets, posing a challenge in experimental selection. Consequently, this research was designed to encompass both ROS and antioxidant experiments, based on the specificity of the activities of the extracts.

### Structural characterization

#### Fourier transform infrared spectroscopy

**Fig. 6:**
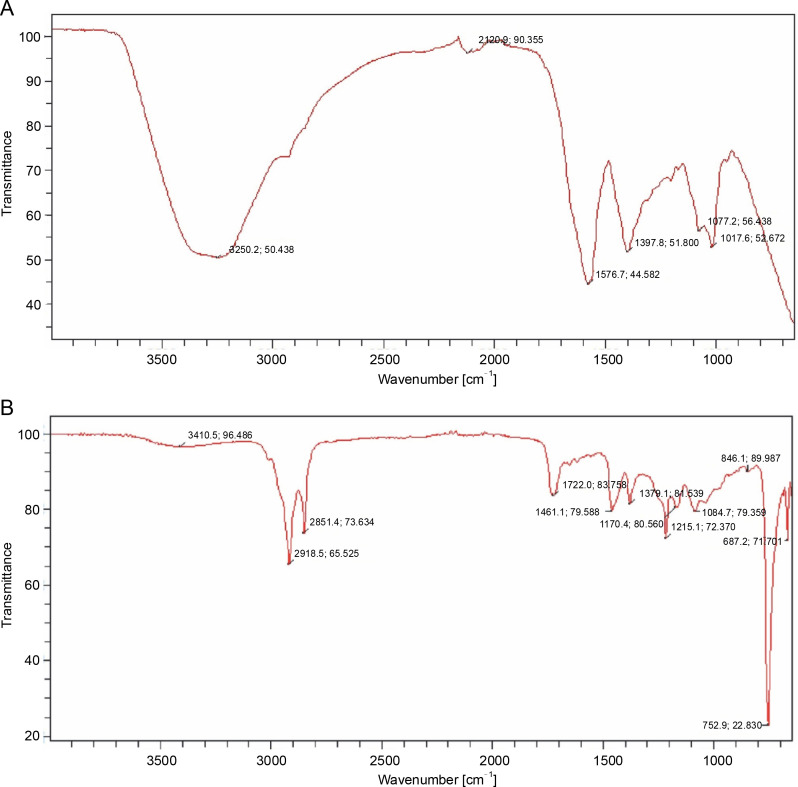
FTIR of the PE (A) and NPE (B) of *Bryophyllum pinnatum* leaves

The FTIR spectra results confirmed the presence of various functional groups in the PE of *B. pinnatum* leaves. Peaks at 3250.2/cm (alcohols, phenols), 2120.9/cm (alkanes), 1576.7/cm (alkenes), 1397.8/cm (aromatics), 1077.2/cm (aliphatic amines), and 1017.6/cm (aliphatic amines) confirmed the presence of these functional groups (Fig. 6A).

Similarly, the FTIR spectra from the NPE of *B. pinnatum* exhibited functional groups with peaks at 3410.5/cm (alcohols, phenols), 2918.5, 2851.4/cm (alkanes), 1722.0/cm (amines), 1461.1/cm (alkanes), 1379.1/cm (alcohols, carboxylic acids, esters, ethers), 1215.1/cm (alkanes), 1084.7/cm (aliphatic amines), 846.1/cm (aromatics), and 667.2/cm (alkyl halides) (Fig. 6B).

The results showed the presence of O–H stretching, C–N stretching, and C=O stretching vibrations, suggesting the presence of carbonyl groups in the extracts. Thus, based on the characterization of fingerprint peak positions, shapes, and intensities, the primary functional groups in the extracts can be easily discerned and characterized (Gomathi et al., 2014). The peaks in the range of 1600–800/cm were assigned to C=O stretching (lipids), whereas 1600–720/cm was assigned to the amide band region of tissue protein (Gomathi et al., 2014). Studies on the structure–activity relationship of certain phenolic compounds, such as tannins, flavonoids, and phenolic acids, suggests that the presence of methoxy and phenolic hydroxyl groups can enhance their antioxidant activities (Jinxiang et al., 2020). Furthermore, the presence of a free hydroxyl group on an aromatic ring is considered indicative of antioxidant activity. The FRS and antioxidant activity of phenolics primarily depend on the number and position of hydrogen-donating hydroxyl groups on the aromatic ring of phenolic molecules (Gomathi et al., 2014; Jinxiang et al., 2020). Functional groups like O–H are ubiquitous in all phenolic compounds, while C–N is a common feature of all alkaloids. The presence of O–H and C–N groups confirms the presence of phenolic compounds and alkaloids in both PE and NPE of *B. pinnatum*, suggesting viable antioxidant activity.

The presence of carboxylic acid, amino acids, alkenes, nitrates, ethers, organic halogen compounds, and carbohydrates in plant material is shown by a more intense band occurring at different frequencies, depicting O–H/N–H, C–H, and C–CHO skeletal vibrations (Muruganantham et al., 2009; Ragavendran, 2011; Gomathi et al., 2014). The presence of carboxylic acid in both PE and NPE of *B. pinnatum* indicated that the assayed plant could be a significant pharmaceutical product for treating ulcers, jaundice, headaches, fever, liver pain, edema, and rheumatic joint pains (Gomathi et al., 2014). The results, depicted in Figure 6, showed that the plant extract is abundant in amides and amino acids. The absence of absorbance in the region 2220–2260/cm indicates the absence of cyanide groups in the assayed plant (Ragavendran et al., 2011; Gomathi et al., 2014), potentially suggesting nontoxicity.

The antioxidant activity of secondary metabolites in the assayed plant extracts was correlated with their functional groups using FTIR analysis. Through FTIR, comprehensive structural descriptions and chemical information about constituents in the assayed plant were obtained. Therefore, the FTIR results showed the presence of different antioxidant-based functional groups in both PE and NPE of *B. pinnatum* leaves.

#### Gas chromatography and mass spectroscopy

This spectroscopic study was conducted using GCMS analysis, which is one of the most widely used techniques for separating plant metabolites. The compounds identified in the PE and NPE of *B. pinnatum* are shown in Table 4 and Table 5, respectively. This study revealed the presence of various secondary metabolites, such as flavonoids, tannins, phenols, alkaloids, anthraquinone, steroids, terpenoids, phenols, glycosides, and saponins. These bioactive phytoconstituents could be responsible for the observed therapeutic activity of various extracts of *B. pinnatum*.

**Table 4:** Compounds identified from the PE of *Bryophyllum pinnatum* leaf using GCMS analysis

Retention time	Peak area	Library/ID	Reference number	CAS number	Spectral match quality
11.0425	0.2117	Oxalic acid, dodecyl 3,5-difluorophenyl ester	220479	1000309-67-7	55
11.3875	0.1726	Cyclotetradecane	61849	000295-17-0	80
11.4276	0.1038	Heptafluorobutyric acid, pentadecyl ester	248207	959261-23-5	46
11.7394	0.7246	4-Tetradecyne	59854	060212-33-1	66
12.1181	0.9465	9-Eicosenoic acid, (Z)-	169286	029204-02-2	60
12.3156	1.1888	Cetene	87833	000629-73-2	91
12.4157	0.2007	5-Tetradecene, (Z)-	61856	041446-62-2	89
12.7666	2.2159	7,11-Hexadecadienal	98680	1000130-85-7	70
12.8261	0.5946	cis-11-Hexadecenal	100562	053939-28-9	89
13.1249	7.2367	9-Octadecene, (E)-	113637	007206-25-9	95
13.344	4.3526	Hexadecane	89838	000544-76-3	96
15.7278	0.37	Pentadecane, 2,6,10,14-tetramethyl-	128856	001921-70-6	98
16.9475	0.3744	Cyclohexadecane	87836	000295-65-8	93
17.0796	0.5372	1-Dodecanol, 2-octyl-	158035	005333-42-6	60
17.5264	8.2354	1-Octadecene	113634	000112-88-9	99
17.9181	15.6447	Tritetracontane	273205	007098-21-7	94
18.7907	0.4289	Undecane, 2-cyclohexyl-	100622	013151-77-4	55
18.9506	0.8331	9-Heptadecanone	115513	000540-08-9	99
20.0611	1.7729	Hexadecanoic acid, methyl ester	130813	000112-39-0	98
20.2932	0.3271	Dibutyl phthalate	138056	000084-74-2	64
20.4569	0.5115	Heptadecyl heptafluorobutyrate	256997	959085-66-6	78
20.64	0.7674	Pentadecafluorooctanoic acid, heptadecyl ester	274382	1000406-04-7	78
20.8738	1.0267	Cyclopentadecane	74572	000295-48-7	91
21.7143	7.5731	9-Eicosene, (E)-	140276	074685-29-3	93
23.1252	0.2334	9,12-Octadecadienoic acid, methyl ester	153873	002462-85-3	99
23.2984	1.1222	11-Octadecenoic acid, methyl ester	155737	052380-33-3	99
23.8914	0.1226	Methyl stearate	157884	000112-61-8	95
26.4361	0.1211	1,19-Eicosadiene	138504	014811-95-1	58
26.575	0.1416	Fumaric acid, cis-hex-3-enyl hexadecyl ester	247784	1000348-87-1	46
28.6495	0.1449	Cyclotetracosane	194001	000297-03-0	99
29.8794	6.6881	1-Docosene	167463	001599-67-3	99
29.9774	0.1346	1,1 :4 ,1*"*-Tercyclohexane	109947	001795-19-3	64
30.5382	4.4943	Diisooctyl phthalate	233366	000131-20-4	91
30.7995	16.3975	3-Eicosene, (E)-	140277	074685-33-9	99
30.9419	1.4327	Erucic acid	195585	000112-86-7	93
31.115	3.4922	Oleic Acid	142069	000112-80-1	91
31.3752	1.4305	9-Tricosene, (Z)-	180806	027519-02-4	90
31.6721	3.2286	9-Octadecenoic acid (Z)-, 2,3-dihydroxypropyl ester	210562	000111-03-5	89
31.7797	4.465	5-Eicosene, (E)-	140275	074685-30-6	95

**Table 5:** Compounds identified from the NPE of *Bryophyllum pinnatum* leaf using GCMS analysis

Retention time	Peak area	Library/ID	Reference number	CAS number	Spectral match quality
17.509	0.3031	17-Pentatriacontene	265113	006971-40-0	87
17.6971	0.1618	Carbonic acid, octadecyl prop-1-en-2-yl ester	209053	1000383-11-5	58
18.9768	0.3316	Eicosyl octyl ether	243191	1000406-38-8	49
19.4046	0.6115	2-Octynoic acid, methyl ester	28541	000111-12-6	38
19.7076	0.1106	Cyclotridecane	49687	000295-02-3	74
20.0652	4.2923	Pentadecanoic acid, 14-methyl-, methyl ester	130843	005129-60-2	97
21.5259	0.6029	Trichloroacetic acid, pentadecyl ester	221903	074339-53-0	93
21.6713	0.3578	7-Hexadecenal, (Z)-	100566	056797-40-1	62
22.2116	1.8153	9-Octadecenoic acid, (E)-	142088	000112-79-8	72
22.8113	3.0989	Undecanoic acid, 10-bromo-	124097	018294-93-4	58
23.114	8.9796	9,12-Octadecadienoic acid, methyl ester	153873	002462-85-3	99
23.2719	19.2819	11-Octadecenoic acid, methyl ester	155737	052380-33-3	99
23.8768	4.1406	Heptadecanoic acid, 15-methyl-, methyl ester	157952	054833-55-5	95
25.2397	17.2066	Z,Z-6,13-Octadecadien-1-ol acetate	167383	1000131-07-0	80
26.001	10.2243	Oleic Acid	142070	000112-80-1	95
26.2701	8.2674	7,11-Hexadecadienal	98680	1000130-85-7	87
26.6171	10.2561	12-Methyl-E,E-2,13-octadecadien-1-ol	140258	1000130-90-4	56
27.0864	4.238	cis-13-Octadecenoic acid	142083	013126-39-1	60
27.38	3.3145	9-Tetradecenal, (Z)-	74488	053939-27-8	93
30.4934	0.9576	Diisooctyl phthalate	233366	000131-20-4	58
34.8112	0.4519	1,5,9-Undecatriene, 2,6,10-trimethyl-, (Z)-	57864	062951-96-6	74
38.3657	0.9957	3,6-Dimethyl-5-oxo-1,2,3,5-tetrahydroimidazo[1,2-a]pyrimidine	35878	058910-42-2	70

**Fig. 7:**
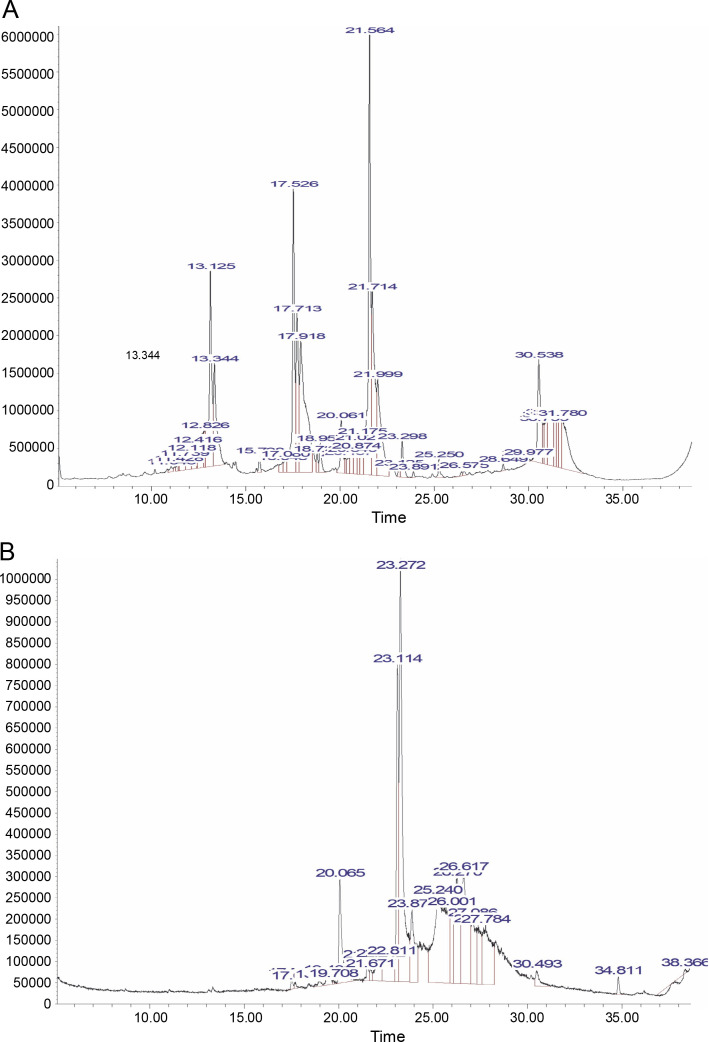
GCMS spectra of the PE (A) and NPE (B) of *Bryophyllum pinnatum* leaf

Figure 7 also depicted the GCMS spectra of the PE (Fig. 7A) and NPE (Fig. 7B) of *B. pinnatum*. A total of thirty-nine (39) compounds were identified in the PE, while twenty-two (22) compounds were identified in the NPE. Notably, oleic acid, 7,11-hexadecadienal, and diisooctyl phthalate were identified in both the PE and NPE. The presence of these phytochemicals, even in trace amounts, contributes to the antioxidant and radical scavenging potentials of *B. pinnatum* leaves. Additionally, pyrimidine was identified in the NPE, while 3-eicosene, 5-eicosene, and 9-eicosene were identified in the PE.

The GCMS investigation of *B. pinnatum* leaves revealed the presence of phytochemical compounds with potential bioactivity, which could contribute to its medicinal properties (Fürer et al., 2016; Yadav et al., 2021). Mbachu et al. (2019) also identified 5-eicosene among the bioactive compounds in the essential oil of *B. pinnatum*. Fatty acid methyl esters (FAME) and fatty acids (FA) are known to possess low antioxidant activity (Pinto et al., 2017; Miya et al., 2023). However, a synergy in their antioxidant activity may explain the higher antioxidant activity of the PE compared to AA, as seen in Figure 2, and the observed antioxidant activity of FA and FAME is evident in both the PE and NPE. FAs can protect against oxidative stress-related diseases by exerting antioxidant roles (Rydlewski et al., 2017). Additionally, oleic acid has demonstrated antioxidant benefits and is well-known for its role in lowering the risk of ROS-induced pathologies and enhancing the immune system (Rydlewski et al., 2017). Recent studies further support the findings on the antioxidant and radical scavenging activity of FAs and FAME (Santos et al., 2024), while research confirms that *B. pinnatum* contains phytochemicals of pharmaceutical and pharmacological relevance (Mbachu et al., 2019).

The functional groups and structures of compounds responsible for the observed antioxidant and FRS activity of the studied extracts were evaluated using FTIR and GCMS analysis, respectively. According to the structure–activity relationship (Jinxiang et al., 2020), the antioxidant and FRS activity of compounds present in *B. pinnatum* is anticipated to correlate with its structure.

## Conclusions

The claims regarding the effect of extracting solvents on the antioxidant and FRS activities of plant extracts find support in the higher antioxidant and FRS potential of the PE compared to the NPE. In this regard, *B. pinnatum* polar extracts emerge as favored strong antioxidant agents for treating diseases associated with oxidative stress. Considering FRAP, DPPH, reducing power, HRSA, and phosphomolybdenum assays, both the PE and NPE of *B. pinnatum* showed antioxidant and FRS properties, indicating the presence of bioactive secondary metabolites with medicinal value in this work. The antioxidant and FRS activities across the extracts generally display a substantial positive correlation, as indicated by the Pearson correlation analysis. The FTIR spectra reveal the existence of bioactive functional groups associated with the plant’s antioxidant-based therapeutic qualities. Furthermore, the antioxidant capacity of these extracts is further supported by the presence of substances known for their antioxidant properties, such as FA and FAME, identified through GCMS analysis. Therefore, our results offer a fresh viewpoint on how to comprehend *B. pinnatum’s* antioxidant and FRS actions. Therefore, to better understand the antioxidant processes of the crude extracts and the extracted phytochemicals, future studies should adopt an *ex vivo*, *in silico*, and *in vivo* approach.

## Conflict of interest

The authors declare no conflict of interest

## References

[cit0001] Abdulrahman M.D. (2022) Chemical composition of Bryophyllum pinnatum (Lam.) Oken. Indonesian J. Pharm. 33(2): 193–199. 10.22146/ijp.2528

[cit0002] Akacha L., Dikko J., Khan M., Anyam J., Igoli J. (2016) Phytochemical screening and antimicrobial activity of Bryophyllum pinnatum extracts. Br. Biotechnol. J. 16(2): 1–8. 10.9734/BBJ/2016/28905

[cit0003] Al-Snafi A.E. (2013) The chemical constituents and pharmacological effects of Bryophyllum calycinum. A review. Int. J. Pharm. Sci. Res. 12: 171.

[cit0004] Amarowicz R. (2007) Tannins: the new natural antioxidant? Eur. J. Lipid Sci. Technol. 109(6): 549–551. 10.1002/ejlt.200700145

[cit0005] Amiri H. (2021) Volatile constituents and antioxidant activity of flowers, stems and leaves of Nasturtium officinale R. Br. Nat. Prod. Res. 26(2): 109–115. 10.1080/14786419.2010.534998 21815727

[cit0006] Asiwe E., Igwe U., Iheanacho K.M.E., Onyeocha I.O., Onwuliri V.A. (2021) Antioxidant and free radical scavenging properties of ethyl acetate fractions of Persea Americana and Bryophyllum pinnatum leaf. Trop. J. Nat. Prod. Res. 5(8): 1486–1492. 10.26538/tjnpr/v5i8.26

[cit0007] Auwal S.M., Saka S., Mairiga A.I., Sanda A.K., Shuaibu, A., Ibrahim A. (2014) Preliminary phytochemical and elemental analysis of aqueous extract and fractionated pod extract of Acacia nilotica (Thorn mimosa). Vet. Res. Forum. 5(2): 95–100. PMID: 25568701 25568701 PMC4279630

[cit0008] Babatunde J.O., Nosarieme O.A., Oyedotun M.O., Boyede O. (2023) In vitro evaluation of the influence of extraction solvents on antioxidant and anti-inflamatory potential of dried leaf of Bryophyllum pinnatum Lam. Contemp. Agric. 72(1–2): 75–81. 10.2478/contagri-2023-0010

[cit0009] Bhandari R., Gyawali S., Aryal N., Gaire D., Paudyal K., Panta A., Panth P., Rokaya R.K., Aryal P., Pandey J (2021) Evaluation of phytochemical, antioxidant, and memory-enhancing activity of Garuga pinnata Roxb. Bark and Bryophyllum pinnatum (Lam) Oken. Leaves. Sci. World J. 6649574: 1–7. 10.1155/2021/6649574 PMC8096568 33994883

[cit0010] Brand-Williams W., Cuvelies M.E., Berset C. (1995) Use of a free radical method to evaluate antioxidant activity. LWT – Food Sci. Technol. 28: 25–30. 10.1016/S0023-6438(95)80008-5

[cit0011] Campbell M.J. (2021) *Statistics at square one*. 12th edition. Wiley-Blackwell.

[cit0012] Chen Y., Miao Y, Huang L., Li. J., Sun H., Zhao Y, Yang J., Zhou W. (2014) Antioxidant activities of saponins extracted from Radix Trichosanthis: an in vivo and in vitro evaluation. BMC Complement. Altern. Med. 14: 86. 10.1186/1472-6882-14-86 24597831 PMC3973866

[cit0013] Fürer K., Simões-Wüst A.P., Mandach U., Hamburger M., Potterat O. (2016) Bryophyllum pinnatum and related species used in anthroposophic medicine: constituents, pharmacological activities, and clinical efficacy. Planta Med. 82: 930–941. 10.1055/s-0042-106727 27220081

[cit0014] Gutierrez-del-Rio I., Lopez-Ibanez S., Fernandez-Corpas P., Fernandez-Calleja, Tunon-Granda M., Miguelez E.M., Villar C.J., Lombo F. (2021) Terpenoids and polyphenols as natural antioxidant agents in food preservation. Antioxidants 10(8): 1264. 10.339/antiox10081264 34439512 PMC8389302

[cit0015] Gutteridge J.M., Halliwell B. (2010) Antioxidants: Molecules, medicines, and myths. Biochem. Biophy. Res. Commun. 393(4): 561–564. 10.1016/j.bbrc.2010.02.071 20171167

[cit0016] Herb M., Schramm M. (2021) Functions of ROS in macrophages and antimicrobial immunity. Antioxidant 10(313): 1–39. 10.3390/antiox10020313 PMC7923022 33669824

[cit0017] Ijoma K.I., Ajiwe V.I.E., Ndubuisi J.O. (2022) Evidence based preferential in vitro antisickling mechanism of three native Nigerian plants used in the management of sickle cell disease. Malaysian J. Biochem. Mol. Biol. 25(3): 9–17.

[cit0018] Ijoma K.I., Ajiwe V.I.E., Odinma S.C. (2023) The organic extracts from the leaves of Ficus thonningii Blume, Jatropha tanjorensis J.L Ellis and Saroja and Justicia carnea Lindley as potential nutraceutical antioxidants and functional foods. Trends Phytochem. Res. 7(1): 76–85. 10.30495/TPR.2023.1977670.1318

[cit0019] Imaobong E.D., Ekemini I.A., Edidiong C.U. (2020) Phytochemical evaluation, antioxidant and antimicrobial activities of various extracts from leaves and stems of Bryophyllum pinnatum. Nepal J. Biotechnol. 8(1): 17–28. 10.3126/njb.v8i1.30206

[cit0020] Jin M., Cai Y.X., Li J.R., Zhao H. (1996) 1,10-phenanthrolineFe^2+^ oxidative assay of hydroxyl radical produced by H_2_O_2_/Fe^2+^. Prog. Biochem. Biophys. 23(6): 553–555.

[cit0021] Jinxiang C., Jing Y., Lanlan M., Jun L., Nasir S., Chan K.K. (2020) Structure-antioxidant activity relationship of methoxy, phenolic hydroxyl, and carboxylic acid groups of phenolic acids. Sci. Rep. 10: 2611. 10.1038/s41598-020-59451-z 32054964 PMC7018807

[cit0022] Kassia M.F.P., Ana C., Thiago A.M.V., Adam M., Raul B.H., Simone G., Mary U.N., Su G. (2022) The psychoactive effects of Bryophyllum pinnatum (Lam.) Oken leaves in young zebrafish. Plos ONE 17(3): e0264987. 10.1371/journal.pone.0264987 35263358 PMC8906576

[cit0023] Majaz Q., Nazim S., Shaikh S., Gomase P., Choudhari A. (2011) Phytochemical analysis of chloroform extract of roots of Kalanchoe pinnata by HPLC and GCMS. Int. J. Pharm. Sci. Res. 2(7): 1693–1699. 10.13040/IJPSR.0975-8232.2(7).1693-99

[cit0024] Mbachu K.A., Ibok M.G., Adeniyi-Akee M.A., Ajala O.E. (2019) Chemical compositions and antioxidant activity of leaf and stem essential oils of Bryophyllum pinnatum (lam.) Kurz. GSC Biol. Pharm. Sci. 9(2): 57–64. 10.30574/gscbps.2019.9.2.0184

[cit0025] Miya G.M., Oriola A.O., Payne B., Cuyler M., Lall N., Oyedeji A.O. (2023) Steroids and fatty acid esters from Cyperus sexangularis Leaf and their antioxidant, anti-inflammatory, and anti-elastase properties. Molecules 28(8): 3434. 10.3390/molecules28083434 37110668 PMC10141076

[cit0026] Mooradian A.D. (1993) Antioxidant properties of steroids. J. Steroid Biochem. Mol. Biol. 45(6): 509–511. 10.1016/0960-0760(93)90166-t 8518206

[cit0027] Muruganantham S., Anbalagan G., Ramamurthy N. (2009) FTIR and SEM-EDS comparative analysis of medicinal plants, Eclipta alba Hassk and Eclipta prostate Linn. Romanian J. Biophys. 19(4): 285–294.

[cit0028] Najafabad A.M., Jamai R. (2014) Free radical scavenging capacity and antioxidant activity of metanolic and ethanolic extract of plum (Prunus domestica L.) in both fresh and dried sample. Avicenna J. Phytomed. 4(5): 343–352. PMCID: 25386397 PMC4224712

[cit0029] Nayak B.S., Marshall J.R., Isitor G. (2010) Wound healing potential of ethanolic extract of Kalanchoe pinnata Lam. Leaf – a preliminary study. Indian J. Exp. Biol., 48(6): 572–576. PMID: 20882759 20882759

[cit0030] Nguelefack T.B., Nana P., Atsamo A.D., Dimo T., Watcho P., Dongmo A.B., Kamanyi A. (2006) Analgesic and anticonvulsant effects of extracts from the leaves of Kalanchoe crenata (Andrews) Haworth (Crassulaceae). J. Ethnopharmacol. 106(1): 70–75. 10.1016/j.jep.2005.12.003 16423479

[cit0031] Okele F.O., Wokem G.N., Nwokah E.G. (2019) Phytochemical and antimicrobial activities of Bryophyllum pinnatum and Vernonia amygdaline leaves extracts on selected microbial isolates from wound infection. J. Adv. Microbiol. 15(3): 1–14. 10.9734/jamb/2019/v15i330090

[cit0032] Okwu D.E., Nnamdi F.U. (2011) Two novel flavonoids from Bryophyllum pinnatum and their antimicrobial activity. J. Chem. Pharm. Res. 3(2): 1–10.

[cit0033] Oladejo A.A. (2020) The effect of Bryophyllum pinnatum (Lam) Oken (Crassulaceae) extract on enzymes involved in the inflammatory pathway. J. Biomed. Pharm. Sci. 3(4): 1–5. 10.37421/jbps.2020.3.281

[cit0034] Oyaizu M. (1986) Studies on products of browning reaction: Anti-oxidative activities of products of browning reaction prepared from glucosamine. J. J. Nutr. 44: 307–315. 10.5264/eiyogakuzashi.44.307

[cit0035] Pinto M.E.A., Araujo S.G., Morals M.I., Sa N.P., Lima C.M., Rosa C.A., Siqueira E.P., Johann S., Lima L.A.R.S. (2017) Antifungal and antioxidant activity of fatty acid methyl esters from vegetable oils. Ann. Braz. Acad. Sci. 89(3): 1671–1681. 10.1590/0001-3765201720160908 28876392

[cit0036] Poljsak B., Suput D., Milisav I. (2013) Achieving the balance between ROS and antioxidants: when to use the synthetic antioxidants. Oxid. Med. Cell. Longev. 2013: 956792. 10.1155/2013/956792 23738047 PMC3657405

[cit0037] Ragavendran P., Sophia D., Arul Raj C., Gopalakrishnan V.K. (2011) Functional group analysis of various extracts of Aerva lanata (L.) by FTIR spectrum. Pharmacologyonline 1: 358–364.

[cit0038] Rydlewski A.A., de Morais R., Rotta E.M., Claus T., Vagula JM., da Silva MC., Junior O.O.S., Visentainer V. (2017) Bioactive compounds, antioxidant capacity, and Fatty acids in different parts of four unexplored fruits. J. Food Qual. 8401074: 9. 10.1155/2017/8401074

[cit0039] Santos D.L.C., Azevedo L.S., Siqueira E.P., Castro A.H.F., Lima L.A.R. (2024) Chemical characterization, antioxidant activity, and cytotoxicity of fatty acids methyl esters from Handroanthus impetiginosus (Mart. Ex DC.) Matios (Bignoniaceae) seeds. Nat. Prod. Res. 38(4): 619–623. 10.1080/14786419.2023.2179624 38285922

[cit0040] Santos S., Zurfluh L., Mennet M., Potterat O., Mandach U., Hamburger, M. and Simões-Wüst A.P. (2021) Bryophyllum pinnatum compounds inhibit oxytocin-induced signaling pathways in human myometrial cells. Front. Pharmacol. 12(632986): 1–10. 10.3389/fphar.2021.632986 PMC7930719 33679416

[cit0041] Yadav P., Mishra A.K., Singh H. (2021) A brief review on chemistry and biological activities of Bryophyllum pinatum (Lam.) Oken family: crassulaceae. Orient. J. Chem. 37(2): 269–280. 10.13005/ojc/370202

